# Mechanical properties evaluation of metacarpophalangeal joint prosthesis with new titanium-nickel memory alloy: a cadaver study

**DOI:** 10.1186/s12891-023-06859-z

**Published:** 2023-09-15

**Authors:** Minzheng Guo, Baochuang Qi, Jun Li, Xiangwen Shi, Haonan Ni, Hongxin Shi, Junxiao Ren, Xizong Zhou, Tao Ye, Ling Yao, Yongqing Xu, Meichao Zhang, Chuan Li

**Affiliations:** 1 Kunming Medical University, 650500 Kunming, China; 2 Department of Orthopaedic, 920th Hospital of Joint Logistics Support Force, No. 212 Daguan Road, Xishan District, 650118 Kunming, China; 3 Dali University, 671003 Dali, China; 4 Yunnan University of Chinese Medicine, 650500 Kunming, China; 5 Department of Orthopedics, Yanjin County Hospital, 657500 Zhaotong, China; 6 Anatomy department, School of Basic Medical Science, Southern Medical University, No. 1023-1063 Shatai South Road, Baiyun District, 510515 Guangzhou, China

**Keywords:** Metacarpophalangeal joint, Artificial joint, Ni-Ti memory alloy, Mechanical property

## Abstract

**Objective:**

Ni-Ti memory alloys are unusual materials for hard-tissue replacement because of their unique superelasticity, good biocompatibility, high strength, low specific gravity, low magnetism, wear resistance, corrosion resistance and fatigue resistance. The current study aims to evaluate its mechanical properties and provide biomechanical basis for the clinical application of the prosthesis.

**Methods:**

Ten adult metacarpophalangeal joint specimens were randomly divided into a prosthesis group (n = 5, underwent metacarpophalangeal joint prosthesis) and a control group (n = 5, underwent sham operation). Firstly, the axial compression strength was tested with BOSE material testing machine to evaluate its biomechanical strength. Secondly, these specimens were tested for strain changes using BOSE material testing machine and GOM non-contact optical strain measurement system to evaluate the stress changes. Thirdly, fatigue test was performed between groups. Lastly, the mechanical wear of the metacarpophalangeal joint prosthesis was tested with ETK5510 material testing machine to study its mechanical properties.

**Results:**

Axial compression stiffness in the prosthesis group was greater than that in the control group in terms of 30 ° and 60 ° flexion positions (P < 0.05). There was no statistically significant difference between two groups with regards to axial compression stiffness and stress change test (P > 0.05). In the fatigue wear test, the mean mass loss in the prosthesis group’s prosthesis was 17.2 mg and 17.619 mm^3^, respectively. The mean volume wear rate was 0.12%. There was no statistically significant difference in the maximum pull-out force of the metacarpal, phalangeal, and polymer polyethylene pads between the prosthesis group and the control group specimens.

**Conclusions:**

Ni-Ti memory alloy metacarpophalangeal joint prosthesis conforms to the biomechanical characteristics of metacarpophalangeal joints without implants, and the fatigue strength can fully meet the needs of metacarpophalangeal joint activities after joint replacement.

## Introduction

The metacarpophalangeal joint plays a crucial role in the functional activities of the hand. Patients with metacarpal and phalangeal bone injury and joint stiffness due to various reasons can be treated by joint fusion, free metatarsophalangeal joint transplantation and artificial metacarpophalangeal joint replacement [1–4]. Burman [5] first reported the replacement of metacarpophalangeal joint, which has gradually been performed for metacarpophalangeal joint stiffness and pain. Artificial metacarpophalangeal joint replacement, including restricted prosthesis, semi restricted prosthesis and non-restricted prosthesis, is a kind of operation that can reconstruct metacarpophalangeal joint, restore joint range of motion, solve joint pain and maintain the appearance of the hand. Even satisfactory outcomes can be achieved for severe metacarpophalangeal arthritis, it is still not as mature as hip and knee replacement. Due to the relatively complex anatomical structure of the metacarpophalangeal joint as well as the difficulties in selecting prosthetic materials, none of prosthesis has been widely used in clinic [6, 7].

Existing articles have studied the biomechanics of Swanson prosthesis using finite element model and artificial bone, but neither finite element model nor artificial bone can simulate the complex microstructure and various mechanical characteristics of organic human bone [8, 9]. Orthopedic biomechanics is a discipline that applies engineering principles, especially mechanical mechanics principles, to clinical medicine to solve the problems encountered in orthopedic department. It is also a very important branch of biomechanics. With the deepening of biomechanics research, biomechanics has become an important tool in orthopedics clinical and scientific research, and has a profound impact on the design of artificial prosthesis, fracture prevention, treatment, rehabilitation and prognosis, as well as clinical diagnosis and treatment [10, 11]. Therefore, the use of human hand specimens and biomechanical methods to study the biomechanical characteristics of the prosthesis after prosthetic replacement are still needed. Ni-Ti memory alloy has been attracted more attentions in recent half a century, which characterized by high strength, low specific gravity, low magnetism, wear resistance, corrosion resistance, fatigue resistance and non-toxicity [12–14]. This material not only has the same elastic modulus as human bone, but also has good biocompatibility and unique continuous automatic compression function [15, 16].

Therefore, Ni-Ti memory alloy was designed as a new type of metacarpophalangeal joint prosthesis. In the current study, we aim to evaluate biomechanical properties of the prosthesis and provide mechanical basis for its clinical application.

## Methods

### Design of Ni-Ti memory alloy metacarpophalangeal joint prosthesis

**Fig. 1:**
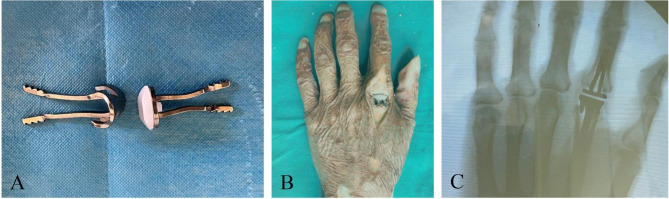
Introduction of Ni-Ti memory alloy prosthesis. **A**, production of Ni-Ti memory alloy prosthesis; **B**, Prosthetic hand specimen; **C**, X-ray after prosthesis implantation

2 mm thick Ni-Ti memory alloy plate was selected as the main material for the prosthesis. High molecular polyethylene was chosen as the gasket material for the distal articulation of the prosthesis. The high molecular polyethylene gasket and the metal contact part of the prosthesis are connected using ultrasonic welding technology. Based on our previous measurement of the anatomical structure of adult metacarpal, phalangeal, and metacarpophalangeal joints, the designed tail length of the prosthesis was set as 25 mm. These were designed as an arc-shaped bend that conforms to the shape of the pulp cavity of the metacarpal and phalangeal bones, by using a one-way memory design to make a support angle of 3° at the tail of the prosthesis. The tail of the prosthesis is designed as an inverted fixed tooth structure to increase resistance to extraction. Deformation and recovery temperatures were 0–4 ℃ and 35–40 ℃, respectively (Fig. 1A). According to the “memory principle” of Ni-Ti memory alloy, the prosthesis handle can be placed into the pulp cavity when the prosthesis is at the deformation temperature. Then, the prosthesis handle can automatically restore to the “mother phase” based on the change of temperature. At the same time, the prosthesis handle will produce a continuous pressure on the pulp cavity wall, so that the prosthesis can be firmly fixed in the pulp cavity (Fig. 1B C). Biomechanical properties were tested to analyze biological properties of the prosthesis, including axial compression stiffness test, strain performance test, fatigue test and mechanical wear test.

### Biomechanical performance test

In order to enable the prosthesis to be used in clinical practice, biomechanical properties were tested, including axial compression stiffness test, strain performance test and fatigue wear test. The cadaver study was approved by the 920th Hospital Ethics Committee of the Joint Logistic Support Force (No.2020-002-01), which consists of five members. All methods were carried out in accordance with relevant guidelines and regulations. Written informed consent was obtained from the the donor.

Adult hand specimens (gender, side no restrictions) were selected in the current study, with an average age of 42 years old, and provided by Henan Daqing Biotechnology Co., LTD, China. Inclusion criteria: the joint capsule and lateral collateral ligament at the metacarpophalangeal joint were intact. Exclusion criteria: anatomical deformities of hands caused by tuberculosis, rheumatism and other reasons in the past. Moreover, patients with fractures and tumors in the hand were also excluded by X-ray examination.

Experimental materials included Ni-Ti memory alloy prosthesis (Lanzhou Ximai Memory Alloy Co., Ltd, China), 3510AT BOSE material testing machine (Tekscan Company, USA), GOM non-contact optical strain measurement system (GOM Company, Germany), surgical instruments, fixtures, self-curing denture acrylic and self-curing denture base (Mechanical Laboratory of Southern Medical University, China). All mechanical experiments were performed at room temperature.

### Axial compression stiffness test

**Fig. 2:**
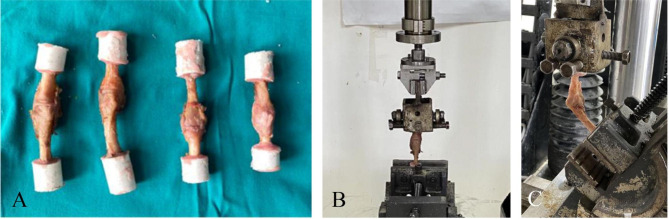
Biomechanical test methods. **A**, Specimen preparation; **B**, The prepared metacarpophalangeal joint specimens were fixed on the BOSE material testing machine in the extended position; **C**, The prepared metacarpophalangeal joint specimens were fixed in the BOSE material testing machine at flexion Angle

Ten hand specimens were randomly divided into prosthesis group and control group according to the digital table method, with 5 sides in each group. In the prosthesis group, 2-5th metacarpophalangeal joint of 5 hand specimens was implanted with new Ni-Ti memory alloy prostheses. Taking the second metacarpophalangeal joint as an example, an arc incision was taken on the dorsum of the second metacarpophalangeal joint. The joint capsule was cut, the metacarpophalangeal joint was exposed, and the cartilage surfaces as well as the bony part of the metacarpal and phalangeal bones were removed. The excision thickness was equal to the width of the prosthesis joint axis. The collateral ligaments at the metacarpophalangeal joint was kept intact during resection. Drill bit was used to make a channel with the same length as the prosthesis handle along the medullary cavity, and then the mushroom drill was used to expand the pulp with almost no destroy to the cortical bone in medullary cavity. The new Ni-Ti memory alloy prosthesis was successfully implanted into the medullary cavity after being placed in 0 ℃ ice water which helps the prosthesis handle closed together. Then 35 ℃ warm water was poured into the pulp cavity to restore the shape of the prosthesis handle. The incised joint capsule was sutured after checking that the implanted prosthesis was not loose and the range of motion of the joint was fine. The metacarpophalangeal joints after prosthesis implantation are shown in Fig. 1B and the X-ray film is shown in Fig. 1C. Similar procedure was performed for the other metacarpophalangeal joints. Then the 2-5th metacarpophalangeal joint of 5 hand specimens were dissociated, retaining intact joint capsule and collateral ligament. Finally, the base of metacarpal bone and proximal phalangeal bone of each dissociated metacarpophalangeal joint were vertically fixed in molds injected with a mixture which was mixed with self-curing denture acrylic and self-curing denture base at a ratio of 5:3. After the self-curing denture acrylic was completely solidified, the preparation of 2-5th metacarpophalangeal joint specimens implanted with new Ni-Ti memory alloy prostheses was completed, as shown in Fig. 2A. In the control group, the specimens were only cut in the same way and then tightly sutured without prostheses implanted. Then the metacarpophalangeal joint was dissociated and fixed by the same method with the prosthesis group.

The specimen was fixed on the BOSE material testing machine in the straight position (Fig. 2B). Before the experiment, to make the specimen in close contact with the BOSE material testing machine and eliminate the error caused by the elastic creep of the ligament, the specimen was preloaded by applying a vertical load at a loading speed of 10 N/S, and keeping for 1 ~ 2 min when the load was up to 100 N [17]. Then, a vertical load of 0 ~ 350 N was applied to the specimen in the straight position at the loading speed of 10 N/S. Axial compression displacement of the specimen was recorded at the load of 50, 100, 150, 200, 250, 300 and 350 N. Axial compression stiffness was further calculated under the vertical load. Similar text was performed for the specimen at different position, including 30° and 60° flexion of metacarpophalangeal joint (Fig. 2C) [18]. Each sample was repeated for 3 times. All specimens were made and fixed by the same researcher, and all data in BOSE material testing machine system were collected and recorded by another researcher. (Computational formula: Axial compression stiffness = axial load/axial displacement; Axial load = maximum loading load - initial load; Axial displacement = maximum load displacement - initial load displacement)

### Strain performance test

**Fig. 3:**
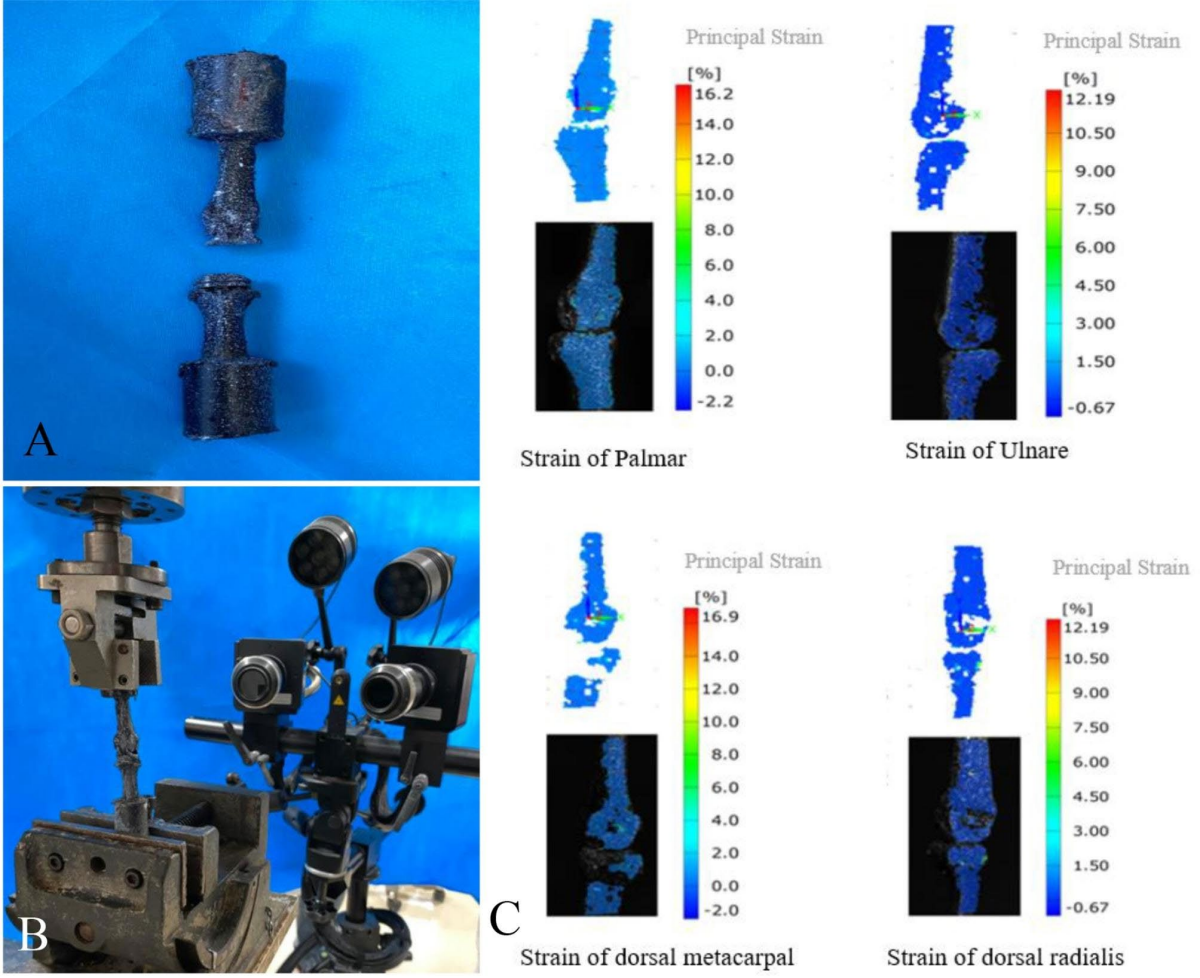
Strain performance test. **A**, Spray speckled specimens; **B**, Measurement of specimens using GOM non-contact optical strain measurement system; **C**, Strain performance diagram obtained from GOM non-contact optical strain measurement system

The second metacarpophalangeal joint of 10 hand specimens was randomly divided into prosthesis group and control group according to the digital table method, with 5 in each group. Second metacarpophalangeal joint of 5 hand specimens were implanted with a new Ni-Ti memory alloy prosthesis as the prosthesis group, with the same surgical method. Soft tissue around the joint were completely removed and only retained the bony part. Subsequently, spray speckle treatment was performed on the specimens (Fig. 3A) [19, 20]. In the control group, 5 specimens of the second metacarpophalangeal joint without implants were set as the control group, which only remove the soft tissue around the joint. Then the same method was used to spray speckle treatment on the specimens.

The two groups of specimens were vertically fixed on the BOSE material testing machine. Before the experiment, the specimen was preloaded with the same method, and then a vertical load of 0 ~ 350 N was applied to the specimen at a loading speed of 10 N/S. The non-contact optical strain gauge was used to measure the specimen at four positions, namely, the palmar, dorsal, ulnar and radial sides (Fig. 3B). Record the strain change at each position when the load is 50, 100, 150, 200, 250, 300 and 350 N, respectively. Strain measurement method: length, width and distance before deformation of the specimen was set as L_0_ and B_0_. The length distance after deformation was set as L and the width distance as B. Grating method was applied to spray speckle on the specimen. The ARAMIS stereo camera in the GOM non-contact optical strain measurement system can use the spray speckle to obtain a large amount of three-dimensional length distance and width distance before and after deformation of the specimen (Fig. 3C), and to further calculate the full field strain. (εy= (L-L0) /L0; εx= (B-B0) /B0).

Strain data is exported through the GOM system, and each specimen is measured 3 times repeatedly. All data collection and specimen production are completed by the same 2 experimenters.

### Fatigue wear test

The Ni-Ti memory alloy metacarpophalangeal joint prosthesis is provided by Lanzhou Ximai Memory Alloy Co., Ltd., and the ETK5510 fatigue testing machine, ultrasonic cleaning machine, vacuum drying box, fixture, etc. are provided by Shanghai Baifan Testing Company; The fatigue wear tests were all completed under indoor constant temperature and humidity conditions, with an ambient temperature of 25 ℃ and a relative humidity of 53%.

Five memory alloy prostheses produced in the same batch (numbered 1–5) were randomly selected using the digital table method. Before experiment, the prostheses were placed in an ultrasonic cleaning machine and shaken with deionized water for 10 min, followed by rinsing with deionized water. Then, a cleaning agent was added to the ultrasonic cleaning machine and shaken again for 10 min before rinsing with deionized water. Furthermore, the prostheses were placed in an ultrasonic cleaning machine containing deionized water and repeatedly shaken and rinsed three times. Each oscillation time is 10 min. After cleaning, the prosthesis is blown dry with inert gas and dried in a vacuum drying oven for 30 min. Subsequently, each prosthesis is weighed three times and recorded. The weighing method follows ISO 14243-2:2016 Implants for surgery-Wear of total knee-joint prostheses-Part 2: Methods of measurement, and the weighing results of each prosthesis are averaged.

**Fig. 4:**
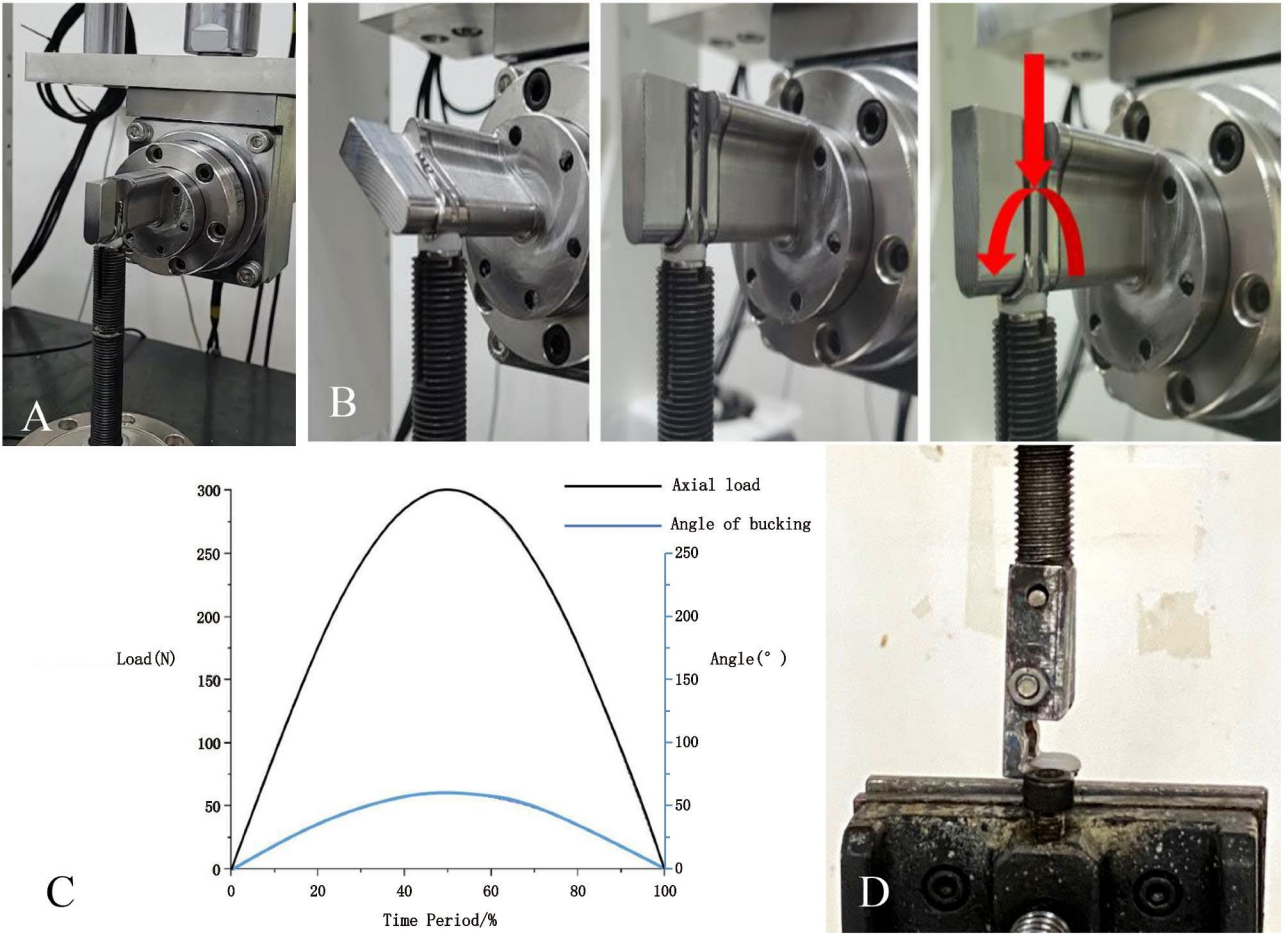
Fatigue wear test. **A**, Memory alloy prosthesis fixed on fatigue testing machine; **B**, Fatigue testing machine simulates flexion and extension activities of metacarpophalangeal joints; **C**, Changes in force/angle action and force/motion action; **D**, Testing the Pullout Resistance of Polymer Polyethylene Gaskets

Specimens were fixed on the ETK5510 material testing machine (Fig. 4A), and applied with 0-300 N axial cyclic load to simulate the flexion and extension movement of metacarpophalangeal joint, with the range of movement of 0–60 ° (the frequency of movement of 1 Hz, and the number of movements of 1,000,000 times)(Fig. 4B). All applied force/angle action and force/motion action follow a fixed periodic change rule (Fig. 4C). After the experiment, all prostheses were cleaned, dried and weighed again, and the mass loss, volume wear and volume wear rate after the experiment were calculated. (Volume wear amount = mass loss/polymer polyethylene density (0.96 mg/m^3^); Volume wear rate = Volume wear amount/Total volume * 100)

Additionally, the polymer polyethylene gasket part of the prosthesis was subjected to pull-out force testing: five prostheses that had undergone fatigue wear testing were assigned to the prosthesis group, and the other five prostheses produced in the same batch that had not undergone fatigue wear testing were assigned to the control group. The prosthesis was fixed on the BOSE material testing machine, with a special fixture to connect the polymer polyethylene gasket part of the prosthesis (Fig. 4D). Then, a vertical upward pulling force was applied to the gasket, with a loading speed of 10 N/S, until the polymer polyethylene gasket is completely pulled out and stop. Record the maximum pulling force at this time.

### Statistical methods

SPSS26.0 statistical software was used to analyze the experimental data. The continuous variable data was expressed in the form of mean ± standard deviation. The difference between the two groups was analyzed by two independent sample t-test, and the difference between the multiple groups was analyzed by one-way ANOVA. P < 0.05 is defined as the difference with statistical significance.

## Result

In the current study, all specimens were fixed on the test machine stably. After the experiment, X-ray fluoroscopy was used. All specimens were free of obvious fractures, and the prosthesis was free of looseness and fracture.

### Axial compression stiffness test

As shown in Table 1, there was no statistically significant difference in axial compression stiffness between two groups in the 0 ° extension position (P > 0.05). In contrast, axial compression stiffness in the prosthesis group was greater than that of the control group at 30 ° and 60 ° flexion (P values < 0.05). Compared within the prosthesis group and the control group, there was no statistically significant difference in axial compression stiffness between different metacarpophalangeal joints at 0 ° extension position and 30 ° and 60 ° flexion position (P > 0.05).

**Table 1:** Comparison of axial compression stiffness of the 2nd to 5th metacarpophalangeal joint specimens of the two groups in the straight position and the flexion position of 30 ° and 60 ° (n=10, N/mm, $\stackrel{-}{\varvec{x}}$±s)

Metacarpophalangeal joint	Prosthesis group			Control group		
	Extending	Flexion 30°	Flexion 60°	Extending	Flexion 30°	Flexion 60°
Second	213.70 ± 9.27	139.56 ± 1.96	122.13 ± 2.76	215.89 ± 12.81	127.75 ± 0.99^a^	107.64 ± 2.07^b^
Third	219.45 ± 4.63	140.58 ± 3.28	129.48 ± 6.56	218.67 ± 5.16	130.55 ± 2.05^a^	110.83 ± 3.72^b^
Fourth	215.35 ± 1.40	141.92 ± 1.37	123.59 ± 2.75	215.85 ± 1.56	130.06 ± 3.42^a^	105.40 ± 0.52^b^
Fifth	215.05 ± 2.45	138.41 ± 0.62	127.17 ± 1.48	217.73 ± 2.16	128.39 ± 0.33^a^	109.50 ± 0.98^b^
T	0.640	1.590	2.229	2.302	1.237	3.438
P	0.611	0.266	0.162	0.154	0.358	0.072

Note: ^a^P<0.05 vs. Flexion 30° in prosthesis group; ^b^P<0.05 vs. Flexion 60° in prosthesis group

### Strain performance test

There was no significant difference between two groups in terms of strain performance test. The strain of metacarpal and phalangeal bones in each group increased with the increase of load. The strain values of the Ni-Ti memory alloy metacarpophalangeal joint prosthesis are consistent with the strain changes of the metacarpophalangeal joint without implants. (Table 2)

**Table 2:** Comparison of stress on metacarpal side between normal group and prosthesis group (n=10, $\stackrel{-}{\varvec{X}}$± S)

Group	Metacarpal side							Phalangeal side						
	50N	100N	150N	200N	250N	300N	350N	50N	100N	150N	200N	250N	300N	350N
Control group	0.16 ± 0.04	0.29 ± 0.10	0.44 ± 0.22	0.58 ± 0.19	0.76 ± 0.36	0.91 ± 0.33	0.96 ± 0.39	0.02 ± 0.03	0.08 ± 0.06	0.16 ± 0.07	0.22 ± 0.06	0.31 ± 0.03	0.37 ± 0.04	0.39± 0.03
Prosthesis group	0.17 ± 0.05	0.41 ± 0.12	0.63 ± 0.18	0.83 ± 0.23	1.07 ± 0.33	1.23 ± 0.39	1.27 ± 0.47	0.06 ± 0.04	0.14 ± 0.09	0.23 ± 0.16	0.26 ± 0.16	0.32 ± 0.18	0.39 ± 0.21	0.41± 0.26
T	-1.097							-0.528						

### Fatigue and mechanical wear test

In mechanical wear testing, all prostheses are stably fixed on the material testing machine. There was no fracture or damage to the prostheses. The average mass loss of the prosthesis is 17.2 mg, the average volume wear is 17.619 mm^3^, and the average volume wear rate is 1.164%, respectively (Table 3). There was no statistically significant difference in the maximum pull-out force of the polymer polyethylene gasket portion between the prosthesis group and the control group (Table 4).

**Table 3:** Mechanical wear test (n = 5)

No.	Mass loss (mg)	Volume wear (mm^3^)	Volume wear rate (%)
1	19.8	20.625	1.35
2	14.2	14.792	0.98
3	16.3	16.978	1.12
4	18.2	17.473	1.16
5	17.5	18.228	1.21
Mean	17.2	17.6192	1.164
Std Dev.	1.88	1.89	0.12

**Table 4:** Comparison of the maximum pull-out force of the polymer polyethylene gasket section between the experimental group and the control group (n=10, N, $\stackrel{-}{\varvec{x}}$±s)

Group	Maximum extraction force of polyethylene gasket (N)
Control group	679.74 ± 1.39
Prosthesis group	669.81 ± 1.16
T	-12.26
P	0.681

## Discussion

The artificial small joint prosthesis has been used for many years, but the shortcomings of the products at this stage are obvious. The design of new joint prosthesis should pursue the following goals: [1] The shape characteristics of the prosthesis can provide sufficient stability for the joint, and reconstruct the balance of the joint, so that the range of motion of the joint after surgery can be as close as possible to the joint without surgery; [2] The prosthesis and bone tissue have good biocompatibility, so that the prosthesis and bone tissue can maintain long-term bone connection; [3] Through the innovation and improvement of prosthesis materials, the bionics and wear resistance of the prosthesis can be improved and fracture around the prosthesis can be avoided [21].

Normal and necessary biomechanical load can maintain the stability of intraarticular environment, but abnormal biomechanical load will accelerate the progression of joint injury and destruction, and aggravate the degree of joint degeneration. With age, long-term biomechanical abnormalities caused by joint trauma, immobilization and chronic cumulative injury can lead to the destruction of joint cartilage and increased joint degenerative changes [22, 23]. Biomechanical methods can be used to analyze the biomechanical characteristics of the prosthesis, and the design of the prosthesis can be gradually optimized, so that the joint implanted with the prosthesis is more in line with the biomechanical characteristics of human body. In the current study, there was no significant difference in the axial compression stiffness of the 2nd to 5th metacarpophalangeal joints between the two groups in the straight position. The axial compression stiffness of prosthesis group was greater than that of control group at 30° and 60° flexion. Possible reasons may be that the polymer polyethylene gasket of the prosthesis has a greater degree of constraint on the joint during flexion of the metacarpophalangeal joint. Therefore, the specimen implanted with the metacarpophalangeal joint prosthesis has a smaller axial displacement under the same load, resulting in a relatively higher axial compression stiffness of the specimen in the prosthesis group. There was no statistically significant difference in axial compression stiffness between different metacarpophalangeal joints in the extension and flexion positions of 30 ° and 60 ° (P values > 0.05), indicating that biomechanical properties of the metacarpophalangeal joint specimens implanted with this prosthesis were consistent with those of metacarpophalangeal joints without implants. In the current study, we also observed that the axial compression stiffness of the specimens in the prosthesis groups and control groups gradually decreased with the increase of joint flexion angle. This phenomenon may be due to the stress concentration phenomenon on the joint surface of the metacarpophalangeal joint without implants at the flexion angle, which reduces the contact area of the joint surface, resulting in an increase in axial compression displacement under the same load. The same phenomenon also occurred in specimens in the prosthesis group. The reason can be explained as the gasket part of the prosthesis is made of ultra-high molecular weight polyethylene material, which is an elastic material, and the gasket is connected to the prosthesis stem through ultrasonic welding. When the load is loaded at the buckling angle, a shear detachment force will be generated on the polyethylene gasket, which will increase its axial displacement and reduce its axial compression stiffness. In the strain test, we found that there was no statistically significant difference in stress changes between two groups, indicating that the strain changes of the specimen implanted with the metacarpophalangeal joint prosthesis were consistent with the strain of the metacarpophalangeal joint without implants. In this study, we also found that the strain of the metacarpal and phalangeal bones in both groups of specimens increased with increasing load. The strain values of each group of specimens measured within the loading range of this experiment are basically normal distribution through statistical analysis, and the strain of metacarpal bone is greater than that of proximal phalanx under the same load. There was also no significant change in the strain of the metacarpal and proximal phalanges in each group when the load was between 300 and 350 N, indicating the so-called plateau period, that is, the elastic deformation of the metacarpal and proximal phalanges has been close to the limit, and the specimen will be destroyed if the load is increased again. The prosthesis also exhibits excellent performance in fatigue testing and mechanical wear testing. It should be noted that the new Ni-Ti memory alloy prosthesis conforms to the biomechanical characteristics of metacarpophalangeal joints without implants, and also has good stability during joint flexion. Its strength and stability can meet the needs of normal activities of metacarpophalangeal joints in daily life.

The operative time of joint replacement can be shorten as much as possible by using this prosthesis we designed. The prosthesis handle can be molded into a shape that can enter the medullary cavity in 0–3 ℃ normal saline. After the prosthesis enters the medullary cavity, 35–40 ℃ normal saline can be poured, and the prosthesis handle can restore the mother phase [24]. At this time, a continuous pressure force will be generated on the medullary cavity wall, and the prosthesis handle can completely and firmly fit the medullary cavity, making complex surgery become a simple installation process, shortening the operation time, reducing the risk of infection. In addition, a large number of studies have shown that Ni-Ti memory alloy has good biocompatibility. The intake of human physiological nickel is 100 ~ 600 ug/d. The World Health Organization stipulates that the minimum daily requirement of human nickel is 20 ug, while the minimum toxic dose is 600 ~ 2500 ug [25]. Therefore, the lower limit of the minimum toxic dose is close to the upper limit of human physiological nickel intake, which indicates that Ni-Ti memory alloy is safe in vivo [26, 27].

Although the prosthesis conforms to the biomechanical characteristics of the metacarpophalangeal joint without implants, and the metacarpophalangeal joint activity after implantation of the prosthesis is also close to normal, Ni-Ti memory alloy also has certain defect. The titanium oxide protective layer can block the release of nickel ions to a certain extent, but the high concentration of nickel in the alloy increases the risk of allergic reactions in individual bodies [28]. Therefore, the development of nickel-titanium free memory alloys has become the focus of attention. In addition, this study only designed two types of prosthesis with reference to the anatomical data of metacarpophalangeal joint, but there are certain anatomical differences in the size of the 2-5th metacarpophalangeal joint and the medullary cavity of the metacarpophalangeal bone and phalangeal bone of the human body. In the later stage, the model of the prosthesis needs to be improved to design different types of prosthesis that meet the anatomical structure of each metacarpophalangeal joint. Finally, this study needs to be verified by a larger sample size, and also needs to be tested by later clinical application. In subsequent studies, we will further carry out animal experiments on the improved prosthesis.

## Conclusions

Eventually, the current study showed that the Ni-Ti memory alloy metacarpophalangeal joint prosthesis conformed to the biomechanical characteristics of metacarpophalangeal joints without implants, and the fatigue strength can fully meet the needs of metacarpophalangeal joint activities after joint replacement.

## Data Availability

All data generated or analyzed during this study are included in this published article.
